# Deep learning models for predicting the survival of patients with hepatocellular carcinoma based on a surveillance, epidemiology, and end results (SEER) database analysis

**DOI:** 10.1038/s41598-024-63531-9

**Published:** 2024-06-09

**Authors:** Shoucheng Wang, Mingyi Shao, Yu Fu, Ruixia Zhao, Yunfei Xing, Liujie Zhang, Yang Xu

**Affiliations:** 1 Department of Gastroenterology, The First Affiliated Hospital of Henan University of Chinese Medicine, The First Clinical Medical College of Henan University of Chinese Medicine, Zhengzhou, 450000 China; 2 Personnel Department, The First Affiliated Hospitalof Henan University of Chinese Medicine, Zhengzhou, 450000 China; 3 Research Department, The First Affiliated Hospital of Henan University of Chinese Medicine, Zhengzhou, 450000 China; 4 Henan Evidence-Based Medicine Center of Traditional Chinese Medicine, The First Affiliated Hospital of Henan University of Chinese Medicine, Zhengzhou, 450000 China

**Keywords:** Primary liver cancer, Predictive model, SEER, Deep learning, Machine learning, Cancer, Oncology, Risk factors

## Abstract

Hepatocellular carcinoma (HCC) is a common malignancy with poor survival and requires long-term follow-up. Hence, we collected information on patients with Primary Hepatocellular Carcinoma in the United States from the Surveillance, Epidemiology, and EndResults (SEER) database. We used this information to establish a deep learning with a multilayer neural network (the NMTLR model) for predicting the survival rate of patients with Primary Hepatocellular Carcinoma. HCC patients pathologically diagnosed between January 2011 and December 2015 in the SEER (Surveillance, Epidemiology, and End Results) database of the National Cancer Institute of the United States were selected as study subjects. We utilized two deep learning-based algorithms (DeepSurv and Neural Multi-Task Logistic Regression [NMTLR]) and a machine learning-based algorithm (Random Survival Forest [RSF]) for model training. A multivariable Cox Proportional Hazards (CoxPH) model was also constructed for comparison. The dataset was randomly divided into a training set and a test set in a 7:3 ratio. The training dataset underwent hyperparameter tuning through 1000 iterations of random search and fivefold cross-validation. Model performance was assessed using the concordance index (C-index), Brier score, and Integrated Brier Score (IBS). The accuracy of predicting 1-year, 3-year, and 5-year survival rates was evaluated using Receiver Operating Characteristic (ROC) curves, calibration plots, and Area Under the Curve (AUC). The primary outcomes were the 1-year, 3-year, and 5-year overall survival rates. Models were developed using DeepSurv, NMTLR, RSF, and Cox Proportional Hazards regression. Model differentiation was evaluated using the C-index, calibration with concordance plots, and risk stratification capability with the log-rank test. The study included 2197 HCC patients, randomly divided into a training cohort (70%, n = 1537) and a testing cohort (30%, n = 660). Clinical characteristics between the two cohorts showed no significant statistical difference (p > 0.05). The deep learning models outperformed both RSF and CoxPH models, with C-indices of 0.735 (NMTLR) and 0.731 (DeepSurv) in the test dataset. The NMTLR model demonstrated enhanced accuracy and well-calibrated survival estimates, achieving an Area Under the Curve (AUC) of 0.824 for 1-year survival predictions, 0.813 for 3-year, and 0.803 for 5-year survival rates. This model's superior calibration and discriminative ability enhance its utility for clinical prognostication in Primary Hepatocellular Carcinoma. We deployed the NMTLR model as a web application for clinical practice. The NMTLR model have potential advantages over traditional linear models in prognostic assessment and treatment recommendations. This novel analytical approach may provide reliable information on individual survival and treatment recommendations for patients with primary liver cancer.

## Introduction

Primary liver cancer, the sixth most common cancer globally in 2020 and the third leading cause of cancer death–accounted for approximately 906,000 new cases and 830,000 deaths. China is a high-incidence country for liver cancer, with the highest number of new and deceased primary liver cancer patients worldwide in 2020^1^. Primary liver cancer includes hepatocellular carcinoma (accounting for about 80% of all primary liver cancer cases), intrahepatic cholangiocarcinoma, and other rare types. Major etiological factors for HCC include chronic infection with Hepatitis B or C viruses (HBV or HCV), exposure to aflatoxins, excessive alcohol consumption, obesity, diabetes, and smoking. Due to population aging, growth, and changes in major etiological factors, the incidence of liver cancer is expected to continue rising in most countries by 2030^2,3^.

Therefore, constructing prognostic models for patients with hepatocellular carcinoma is crucial. Personalized predictive models can better assist clinicians in making treatment decisions or designing clinical trials. Previous studies have used various types of predictive models to forecast the survival of patients with HCC, including the AJCC TNM staging system, logistic regression analysis, and the Cox proportional-hazards model^4–7^. The AJCC TNM staging system is currently the most widely used cancer staging system worldwide. It is mainly based on tumor size, number, lymph node involvement, and distant metastasis to stage patients and predict their prognosis^8^. In these models, nomograms, which use the Cox proportional hazards (CoxPH) model to assess patient prognosis, are common. However, the Cox proportional hazards model assumes that each predictor has the same effect at different follow-up times, ignoring the variability in the impact of prognostic factors on individual patients over time. Additionally, these models adopt linear assumptions and do not consider non-linear analyses in real-world clinical aspects^9,10^. Thus, more accurate models are needed to fit survival data with non-linear functions better.

In recent years, with the rapid development of artificial intelligence technology, AI applications, including in liver diseases, have increased. Deep learning, an emerging field, has been widely applied in the biomedical field. Deep learning algorithms can process a large amount of medical data, such as structured numeric data (e.g., vital signs and lab results), high-dimensional data from multi-omics studies, and digitalized images from various high-resolution radiological and histopathological studies, providing significant technical support for innovative research in the medical field^11^. At the same time, deep learning algorithms have provided more accurate prognostic assessments for cancer patients^12,13^. Some studies found that the RSF model, a regression algorithm based on decision tree ensemble learning, outperforms the Cox proportional hazards (CPH) model regarding differentiation, calibration, clinical utility, and performance^14^. NMTLR is a deep neural network survival analysis model based on a multi-task framework, which introduces a multilayer perceptron (MLP) to increase modeling flexibility. It can predict individual survival and risk functions based on feature vectors without assuming proportional hazards or linear combinations^15^. The DeepSurv model is a deep neural network-based Cox proportional hazards model that can accurately summarize the relationship between patient covariates and their risk of death and provide personalized treatment recommendations for physicians. DeepSurv and NMTLR have the potential to supplement traditional survival analysis methods and become standard methods for physicians to study and recommend personalized treatment plans^16^.

Compared to previous studies, this research utilizes the Surveillance, Epidemiology, and End Results (SEER) database managed by the National Cancer Institute (NCI) to gather detailed clinical data on patients with hepatocellular carcinoma. The SEER database, collecting data from 18 regional cancer registries, covers approximately 28% of the U.S. population. It provides a representative and diverse sample base, enhancing the extrapolation of the research findings. Furthermore, the extensive longitudinal follow-up data within SEER are crucial for analyzing survival trends and assessing the effectiveness of treatment strategies. In this study, we employed the Cox proportional hazards model along with three machine learning models—Random Survival Forests (RSF), Nested Multistate Transition Logistic Regression (NMTLR), and DeepSurv—to develop predictive models for overall survival (OS) in patients with hepatocellular carcinoma. We compared the predictive performance of these models and selected the best-performing model to create an online calculator for real-time use by clinicians, thereby improving the efficiency and accuracy of clinical decision-making. In summary, this study leverages deep learning technologies to process and analyze large-scale cancer data, aiming to provide more accurate survival predictions for patients with hepatocellular carcinoma, thus offering a scientific basis for clinical decision-making.

## Materials and methods

### Study subjects and data source

This retrospective cohort study extracted HCC patients registered in the SEER database of the National Cancer Institute from 2000 to 2018 to construct the model. The SEER database collects information from 18 cancer registries, covering about 28% of the U.S. population. The dataset selected was the SEER Research Plus Data, 18 Registries, Nov 2020 Sub, using SEER*Stat software (version 8.4.1) to extract training cases. Inclusion criteria were: (1) Morphology codes (8170/3–8175/3) according to the International Oncology Code 3rd Edition (ICD-03); (2) Diagnosis year: 2010–2015; (3) Identified as a primary tumor (first malignant primary indicator = yes). Exclusion criteria were: (1) Incomplete follow-up information; (2) Incomplete clinical characteristic factors; (3) Unclear staging and grading; (4) Survival time less than or equal to one month; (5) Age under 18 years.

### Research variables and grouping

This research incorporates a variety of variables for analysis: demographic details such as gender, age, race, marital status, survival status, survival months; tumor characteristics including size, number, histological type, grading, T (AJCC 7th edition), N (AJCC 7th edition), M (AJCC 7th edition), biochemical markers encompassing alpha-fetoprotein (AFP) and liver fibrosis score; and treatment specifics covering surgery, radiotherapy, and chemotherapy. Regarding liver fibrosis, patients are categorized using the Ishak scoring system into two groups: 0–4 (no to moderate fibrosis) and 5–6 (advanced/severe fibrosis). AFP levels are classified into two categories: positive/elevated and negative/normal or within normal range. The selection of these variables is based on their potential significance in predicting the prognosis of patients with hepatocellular carcinoma.

### Data preprocessing

Numeric variables were processed using data standardization methods, and the optimal cutoff values for patient age and tumor size were selected using x-tile software. Other data processing and statistical analysis were performed using R software (version 4.2.3). Continuous variables were represented by mean and standard deviation, categorical variables by percentage and frequency, and group comparisons were made using the chi-square test. All tests were two-sided, with a significance level set at p < 0.05. When two features showed a strong mutual correlation, collinearity emerged. Highly correlated features should be avoided as they increase computational costs and workload and potentially overcomplicate the model. Therefore, we used the cor function in the stats R package to calculate correlations between features. A Pearson correlation value of 0.7 indicated a high degree of collinearity. Additionally, univariate and multivariate Cox regression models were used to assess the importance of potential features.

### Model construction

Python was used for model construction. The primary outcome was the overall survival rate (OS). Four algorithms were selected for training, two based on machine learning (DeepSurv, Neural Multi-Task Logistic Regression [NMTLR], Random Forest [RSF]), and compared with the Cox survival regression model (coxph). The dataset was randomly divided into a training dataset and a test dataset in a 7:3 ratio. Finding the optimal configuration for our model, including network architecture and hyperparameter values, was crucial. We adjusted hyperparameters through 1000 iterations of random search and fivefold cross-validation on the training set. The performance of models with different hyperparameter combinations was assessed using the concordance index (C-index).The difference between the two models’ C-index was tested using Kang’s method^17^. Model accuracy was assessed using the C-index, and we applied the Brier score to represent the mean squared difference between the observed patient state and the predicted survival probability. We also calculated the Integrated Brier Score (IBS) to determine the model's overall performance. Calibration plots were used to calibrate 1-year, 3-year, and 5-year OS, comparing expected and actual survival rates. To assess the time-dependence, sensitivity, and specificity of the model, Receiver Operating Characteristic (ROC) curves were generated, and the Area Under the Curve (AUC) values for 1-year, 3-year, and 5-year survival rates were calculated. To establish the relationship between individual features and model performance, we used a random replacement method to assess the importance of each feature in the test set. First, the model performance was quantified using the concordance index, and then calculations were made using the replaced dataset to evaluate each feature's contribution to model performance. To assess the risk stratification efficacy of the model exhibiting optimal performance, the procedure commences with the calculation of risk probabilities utilizing the algorithm that demonstrated superior efficacy. Optimal threshold values for these probabilities are ascertained through the application of X-tile software. Following this, patients are classified into low, intermediate, and high-risk categories according to the established thresholds. The final phase involves the comparison of survival curves across these risk groups, employing the log-rank test to discern statistically significant differences.

### Model application

The best-performing algorithm was deployed using the Streamlit package in Python to create a web-based interactive tool for practical use.

### Ethics statement

Since the SEER database comprises de-identified patient data that is publicly accessible, the use of this database for our project did not necessitate review by an ethics committee.
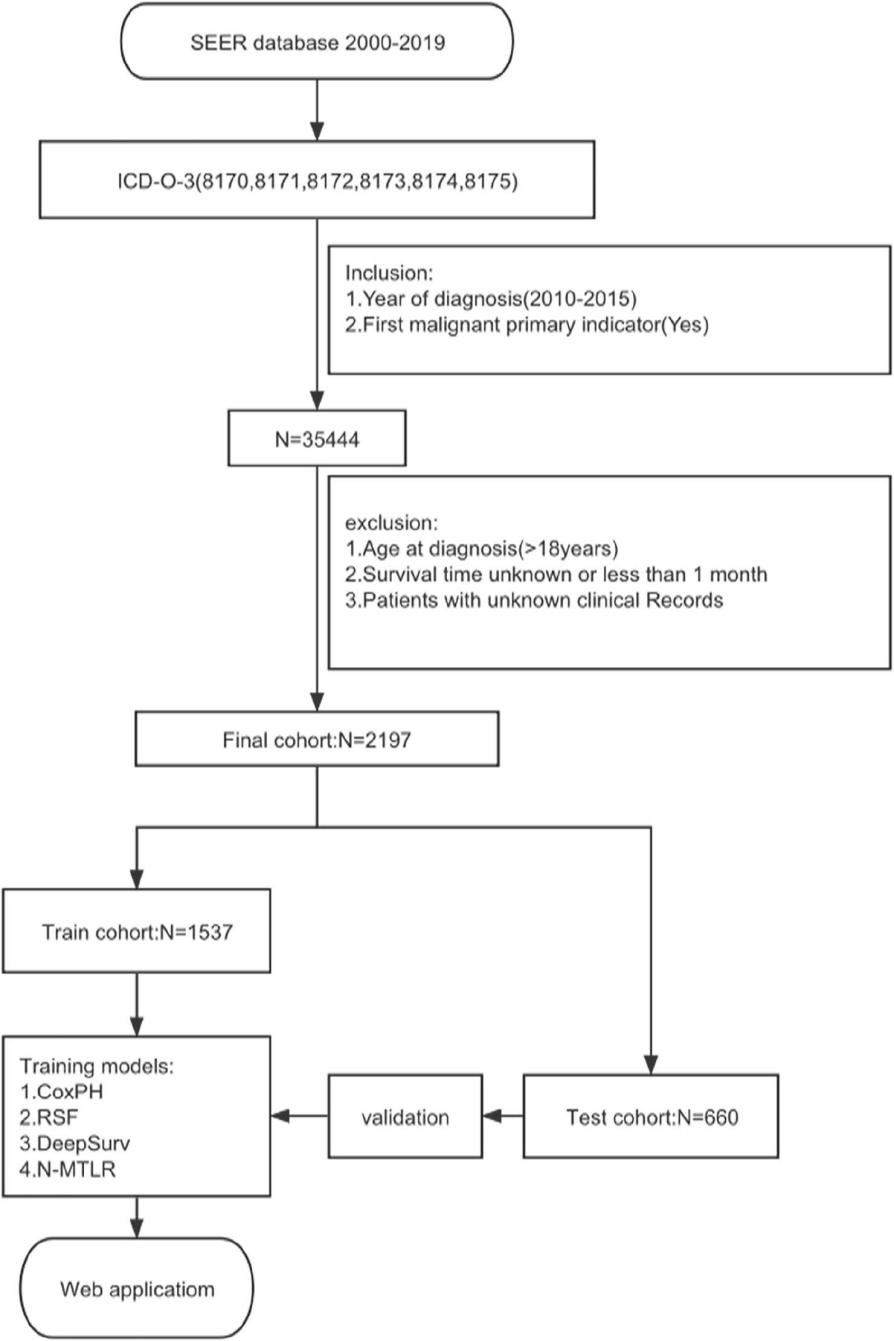


## Results

### Data description

In this study, 35,444 HCC patients were screened from the SEER database between 2010 and 2015, with 2197 patients meeting the criteria for inclusion. Table 1 shows the patients’ main baseline clinical characteristics (eTable 1 in the Supplement). Among the 2197 participants, 70% (n = 1548) were aged 66 years and below, 23% (n = 505) were between 66 and 77 years old, and 6.6% (n = 144) were over 77 years old. Male participants accounted for 78% (n = 1915), while females represented 22% (n = 550). In terms of race, the majority of participants were White, accounting for 66% (n = 1455), followed by Asians or Pacific Islanders at 22% (n = 478), Black individuals at 10% (n = 228), and Native Americans/Alaskan Natives at only 1.6% (n = 36). Regarding marital status, 60% (n = 1319) were married, and the remaining 40% (n = 878) were of other marital statuses. Histologically, most participants (98%, n = 2154) were of type 8170. Additionally, 50% (n = 1104) of the patients were grade II differentiated, 18% (n = 402) were grade III, 1.0% (n = 22) were grade IV, and 30% (n = 669) were grade I. In terms of tumor staging, 48% (n = 1054) of participants were at stage I, 29% (n = 642) at stage II, 16% (n = 344) at stage III, and 7.1% (n = 157) at stage IV. Regarding the TNM classification, 49% (n = 1079) were T1, 31% (n 1 = 677) were T2, 96% (n = 2114) were N0, and 95% (n = 2090) were M0. 66% (n = 1444) of the participants had a positive/elevated AFP. 70% (n = 1532) showed high levels of liver fibrosis. 92% (n = 2012) had a single tumor, while the remaining 8.4% (n = 185) had multiple tumors. 32% (n = 704) underwent lobectomy, 14% (n = 311) underwent local tumor destruction, 34% (n = 753) had no surgery, and 20% (n = 429) underwent wedge or segmental resection. Finally, 2.1% (n = 46) received radiation therapy, with 62% (n = 1352) not receiving chemotherapy and 38% (n = 855) undergoing chemotherapy. The average overall survival (OS) in months for participants was 45 ± 34 months, with 1327 (60%) surviving at the end of follow-up.

**Table 1:** Univariate and multivariate Cox regression analyses of main characteristics.

Characteristic	Overall	Univariate Cox			Multivariate Cox		
	N = 2197^a^	HR^b^	95% CI^b^	p-value	HR^b^	95% CI^b^	p-value
Age				**< 0.001**			**< 0.001**
≤ *66*	1548 (70%)	–	–		–	–	
> *66,* ≤ *77*	505 (23%)	1.32	1.16, 1.49		1.22	1.07, 1.39	
> *77*	144 (6.6%)	2.23	1.85, 2.69		1.67	1.37, 2.04	
Race				** < 0.001**			0.082
American Indian/Alaska Native	36 (1.6%)	–	–		–	–	
Asian or Pacific Islander	478 (22%)	0.71	0.47, 1.08		1.36	0.88, 2.10	
Black	228 (10%)	1.05	0.68, 1.62		1.64	1.05, 2.56	
White	1455 (66%)	0.91	0.61, 1.36		1.47	0.97, 2.24	
Marital_status				** < 0.001**			** < 0.001**
Married	1319 (60%)	–	–		–	–	
Other	878 (40%)	1.38	1.24, 1.54		1.27	1.13, 1.42	
Histological_type				** < 0.001**			**0.002**
8170	2154 (98%)	–	–		–	–	
8171	2 (< 0.1%)	1.94	0.49, 7.78		3.26	0.79, 13.5	
8172	3 (0.1%)	0.00	0.00, Inf		0.00	0.00, Inf	
8173	3 (0.1%)	27.5	8.77, 86.3		7.91	2.46, 25.5	
8174	34 (1.5%)	1.25	0.84, 1.88		1.55	1.02, 2.35	
8175	1 (< 0.1%)	4.97	0.70, 35.4		6.37	0.88, 46.1	
Grade				** < 0.001**			** < 0.001**
Moderately differentiated; Grade II	1104 (50%)	–	–		–	–	
Poorly differentiated; Grade III	402 (18%)	1.55	1.35, 1.79		1.32	1.14, 1.54	
Undifferentiated; anaplastic; Grade IV	22 (1.0%)	1.86	1.17, 2.98		1.36	0.84, 2.19	
Well differentiated; Grade I	669 (30%)	0.95	0.84, 1.08		0.81	0.71, 0.92	
Stage				** < 0.001**			** < 0.001**
I	1054 (48%)	–	–		–	–	
II	642 (29%)	1.23	1.08, 1.41		1.53	0.85, 2.73	
III	344 (16%)	3.26	2.83, 3.77		2.20	1.34, 3.61	
IV	157 (7.1%)	5.59	4.64, 6.74		3.33	1.79, 6.16	
T				** < 0.001**			** < 0.001**
T1	1079 (49%)	–	–		–	–	
T2	677 (31%)	1.25	1.10, 1.43		0.85	0.49, 1.50	
T3a	260 (12%)	2.98	2.54, 3.49		0.79	0.48, 1.28	
T3b	124 (5.6%)	5.24	4.27, 6.43		1.33	0.80, 2.19	
T4	57 (2.6%)	4.64	3.51, 6.13		1.30	0.75, 2.25	
N				** < 0.001**			0.083
N0	2114 (96%)	–	–		–	–	
N1	83 (3.8%)	3.55	2.81, 4.49		0.67	0.42, 1.06	
M				** < 0.001**			0.89
M0	2090 (95%)	–	–		–	–	
M1	107 (4.9%)	4.53	3.68, 5.56		1.04	0.64, 1.67	
AFP				** < 0.001**			**0.050**
Negative/normal; within normal limits	753 (34%)	–	–		–	–	
Positive/elevated	1444 (66%)	1.39	1.23, 1.56		1.13	1.00, 1.28	
Tumor_size				** < 0.001**			** < 0.001**
≤ 62 mm	1629 (74%)	–	–		–	–	
> 62 mm	568 (26%)	2.28	2.04, 2.56		1.74	1.50, 2.01	
Surgery				** < 0.001**			** < 0.001**
Lobectomy	704 (32%)	–	–		–	–	
Local tumor destruction	311 (14%)	1.91	1.59, 2.30		2.32	1.92, 2.81	
No	753 (34%)	4.80	4.15, 5.54		4.25	3.61, 4.99	
Wedge or segmental resection	429 (20%)	1.32	1.10, 1.58		1.29	1.07, 1.56	
Chemotherapy				** < 0.001**			**0.005**
No/Unknown	1352 (62%)	–	–		–	–	
Yes	845 (38%)	1.52	1.37, 1.70		0.84	0.74, 0.95	

Significant values are in bold.

^a^n (%); mean (SD).

^b^*HR* Hazard ratio, *CI* Confidence interval.

### Feature selection

**Figure 1:**
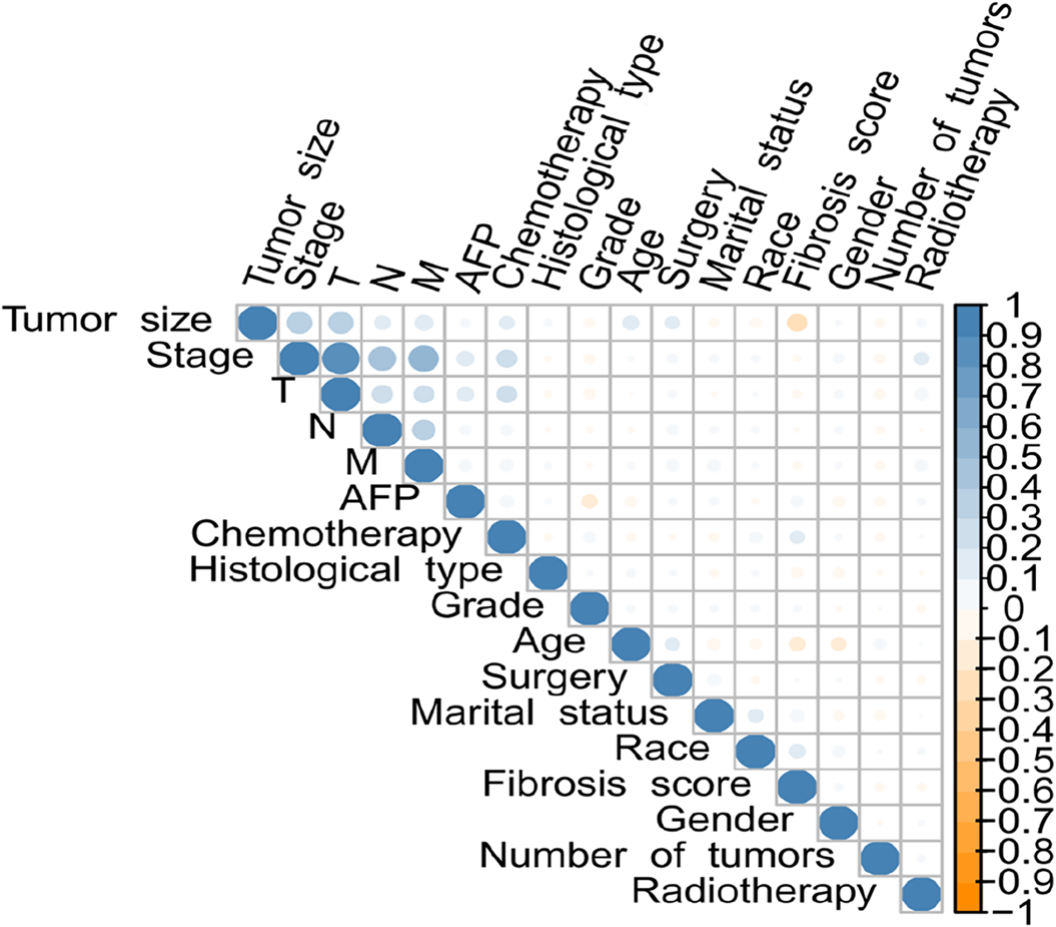
Correlation coeffcients for each pair of variables in the data set.

Following univariate Cox regression analysis, we identified several factors significantly correlated with the survival rate of hepatocellular carcinoma patients (p < 0.05). These factors included age, race, marital status, histological type, tumor grade, tumor stage, T stage, N stage, M stage, alpha-fetoprotein levels, tumor size, type of surgery, and chemotherapy status. These variables all significantly impacted patient survival in the univariate analysis. However, in the multivariate Cox regression analysis, we further confirmed that only age, marital status, histological type, tumor grade, tumor stage, and tumor size were independent factors affecting patient survival (p < 0.05) (Table 1). Additionally, through collinearity analysis, we observed a significant high degree of collinearity between tumor staging (Stage) and the individual stages of T, N, and M (Fig. 1). This phenomenon occurs primarily because the overall tumor stage (Stage) is directly determined based on the results of the TNM assessment. This collinearity suggests the need for cautious handling of these variables during modeling to avoid overfitting and reduced predictive performance. Despite certain variables not being identified as independent predictors in multivariable analysis, we incorporated them into the construction of our deep learning model for several compelling reasons. Firstly, these variables may capture subtle interactions and nonlinear relationships that are not readily apparent in traditional regression models, but can be discerned through more sophisticated modeling techniques such as deep learning. Secondly, including a broader set of variables may enhance the generalizability and robustness of the model across diverse clinical scenarios, allowing it to better account for variations among patient subgroups or treatment conditions. Based on this analysis, we ultimately selected 12 key factors (age, race, marital status, histological type, tumor grade, T stage, N stage, M stage, alpha-fetoprotein, tumor size, type of surgery, chemotherapy) for inclusion in the construction of the predictive model. We divided the dataset into two subsets: a training set containing 1537 samples and a test set containing 660 samples (Table 2). By training and testing the model on these data, we aim to develop a model that can accurately predict the survival rate of hepatocellular carcinoma patients, assisting in clinical decision-making and improving patient prognosis.

**Table 2:** Main characteristic distribution of data in training sets and test sets.

Characteristic	Overall	Train	Test	p-value
	N = 2197^a^	N = 1537^a^	N = 660^a^	
Age (%)				0.094
> 66, ≤ 77	505 (23%)	355 (23%)	150 (23%)	
> 77	144 (6.6%)	112 (7.3%)	32 (4.8%)	
≤ 66	1548 (70%)	1070 (70%)	478 (72%)	
Race (%)				0.470
American Indian/Alaska Native	36 (1.6%)	29 (1.9%)	7 (1.1%)	
Asian or Pacific Islander	478 (22%)	323 (21%)	155 (23%)	
Black	228 (10%)	157 (10%)	71 (11%)	
White	1455 (66%)	1028 (67%)	427 (65%)	
Marital status (%)				0.298
Married	1319 (60%)	924 (60%)	395 (60%)	
Other	878 (40%)	613 (40%)	265 (40%)	
Histological type (%)				0.944
8170	2154 (98%)	1507 (98%)	647 (98%)	
8171	2 (< 0.1%)	2 (0.1%)	0 (0.0%)	
8172	3 (0.1%)	3 (0.2%)	0 (0.0%)	
8173	3 (0.1%)	1 (< 0.1%)	2 (0.3%)	
8174	34 (1.5%)	23 (1.5%)	11 (1.7%)	
8175	1 (< 0.1%)	1 (< 0.1%)	0 (0.0%)	
Grade (%)				0.719
Moderately differentiated; Grade II	1104 (50%)	765 (50%)	339 (51%)	
Poorly differentiated; Grade III	402 (18%)	288 (19%)	114 (17%)	
Undifferentiated; anaplastic; Grade IV	22 (1.0%)	17 (1.1%)	5 (0.8%)	
Well differentiated; Grade I	669 (30%)	467 (30%)	202 (31%)	
T (%)				0.713
T1	1079 (49%)	751 (49%)	328 (50%)	
T2	677 (31%)	484 (31%)	193 (29%)	
T3a	260 (12%)	176 (11%)	84 (13%)	
T3b	124 (5.6%)	84 (5.5%)	40 (6.1%)	
T4	57 (2.6%)	42 (2.7%)	15 (2.3%)	
N (%)				0.726
N0	2114 (96%)	1477 (96%)	637 (97%)	
N1	83 (3.8%)	60 (3.9%)	23 (3.5%)	
M (%)				0.370
M0	2090 (95%)	1452 (94%)	638 (97%)	
M1	107 (4.9%)	85 (5.5%)	22 (3.3%)	
AFP (%)				0.576
Negative/normal; within normal limits	753 (34%)	533 (35%)	220 (33%)	
Positive/elevated	1444 (66%)	1004 (65%)	440 (67%)	
Tumor size (%)				0.387
> 62 mm	568 (26%)	406 (26%)	162 (25%)	
≤ 62 mm	1629 (74%)	1131 (74%)	498 (75%)	
Surgery (%)				0.843
Lobectomy	704 (32%)	485 (32%)	219 (33%)	
Local tumor destruction	311 (14%)	221 (14%)	90 (14%)	
No	753 (34%)	526 (34%)	227 (34%)	
Wedge or segmental resection	429 (20%)	305 (20%)	124 (19%)	
Chemotherapy (%)				0.525
No/unknown	1352 (62%)	953 (62%)	399 (60%)	
Yes	845 (38%)	584 (38%)	261 (40%)	
Survival months (mean (SD))	45 (34)	45 (34)	46 (34)	0.606
Status (%)				0.625
Alive	870 (40%)	603 (39%)	267 (40%)	
Dead	1327 (60%)	934 (61%)	393 (60%)	

^a^n (%); mean (SD).

### Hyperparameter optimization and model comparison results

**Figure 2:**
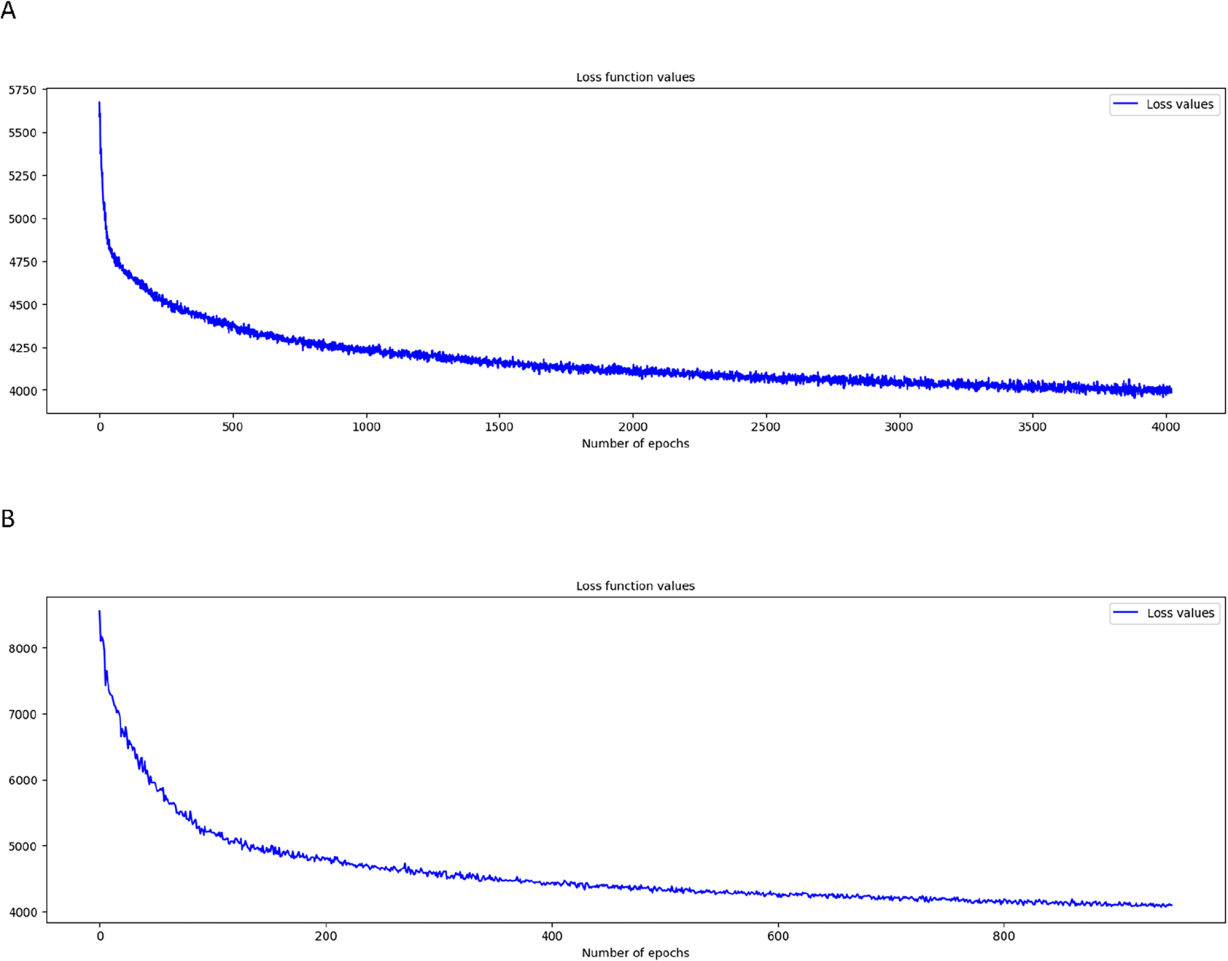
Loss convergence graph for (**A**) DeepSurv, (**B**) neural network multitask logistic regression (N-MTLR) models.

Initially, we conducted fivefold cross-validation on the training set and performed 1000 iterations of random search. Among all these validations, we selected parameters that showed the highest average concordance index (C-index) and identified them as the optimal parameters. Figure 2 displays the loss function graphs for the two deep learning models, NMTLR and DeepSurv. This set of graphs reveals the loss changes of these two models during the training process.

**Figure 3:**
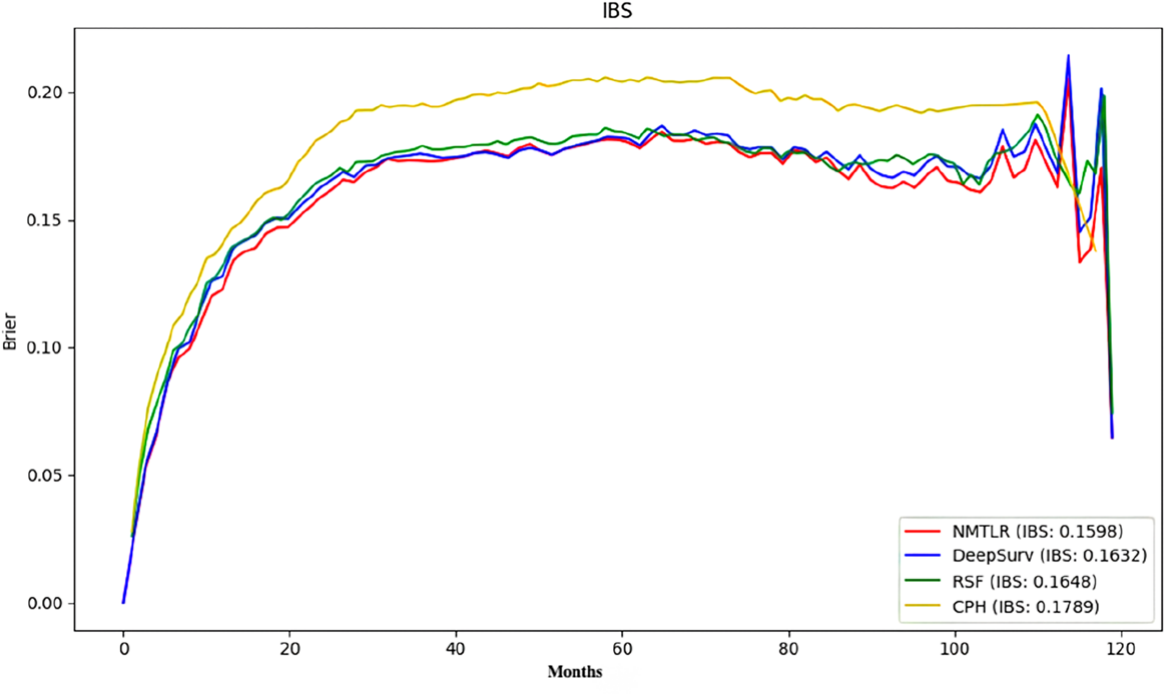
Prediction error curve.

When comparing the machine learning models with the standard Cox Proportional Hazards (CoxPH) model in terms of predictive performance, Table 3 presents the performance of each model on the test set. In our analysis, we employed the log-rank test to compare the concordance indices (C-index) across models. The results indicated that the three machine learning models—DeepSurv, N-MTLR, and RSF—demonstrated significantly superior discriminative ability compared to the standard Cox Proportional Hazards (CoxPH) model (p < 0.01), as detailed in Table 4. Specifically, the C-index for DeepSurv was 0.7317, for NMTLR was 0.7353, and for RSF was 0.7336, compared to only 0.6837 for the standard CoxPH model. Among these three machine learning models, NMTLR had the highest C-index, demonstrating its superiority in predictive performance. Further analysis of the Integrated Brier Score (IBS) for each model revealed that the IBS for the four models were 0.1598 (NMTLR), 0.1632 (DeepSurv), 0.1648 (RSF), and 0.1789 (CoxPH), respectively (Fig. 3). The NMTLR model had the lowest IBS value, indicating its best performance in terms of uncertainty in the predictions. Additionally, there was no significant difference between the C-indices obtained from the training and test sets, suggesting that the NMTLR model has better generalization performance in the face of real-world complex data and can effectively avoid the phenomenon of overfitting.

**Table 3:** Performance of four survival models.

Models	C-index					
	Train	Test	IBS	1-year AUC	3-year AUC	5-year AUC
CoxPH	0.6895	0.6837	0.1789	0.762	0.772	0.737
Deepsurv	**0.7504**	0.7317	0.1632	0.807	0.808	0.800
NMTLR	0.7445	**0.7353**	**0.1598**	**0.824**	**0.813**	**0.803**
RSF	0.7449	0.7336	0.1648	0.812	0.810	0.795

Significant values are in bold.

**Table 4:** Comparative analysis of discriminative ability (C-index) between CoxPH and machine learning models (DeepSurv, N-MTLR, RSF).

Model 1	Model 2	p-value
CoxPH	NMTLR	< 0.01
CoxPH	RSF	< 0.01
CoxPH	DeepSurv	< 0.01

**Figure 4:**
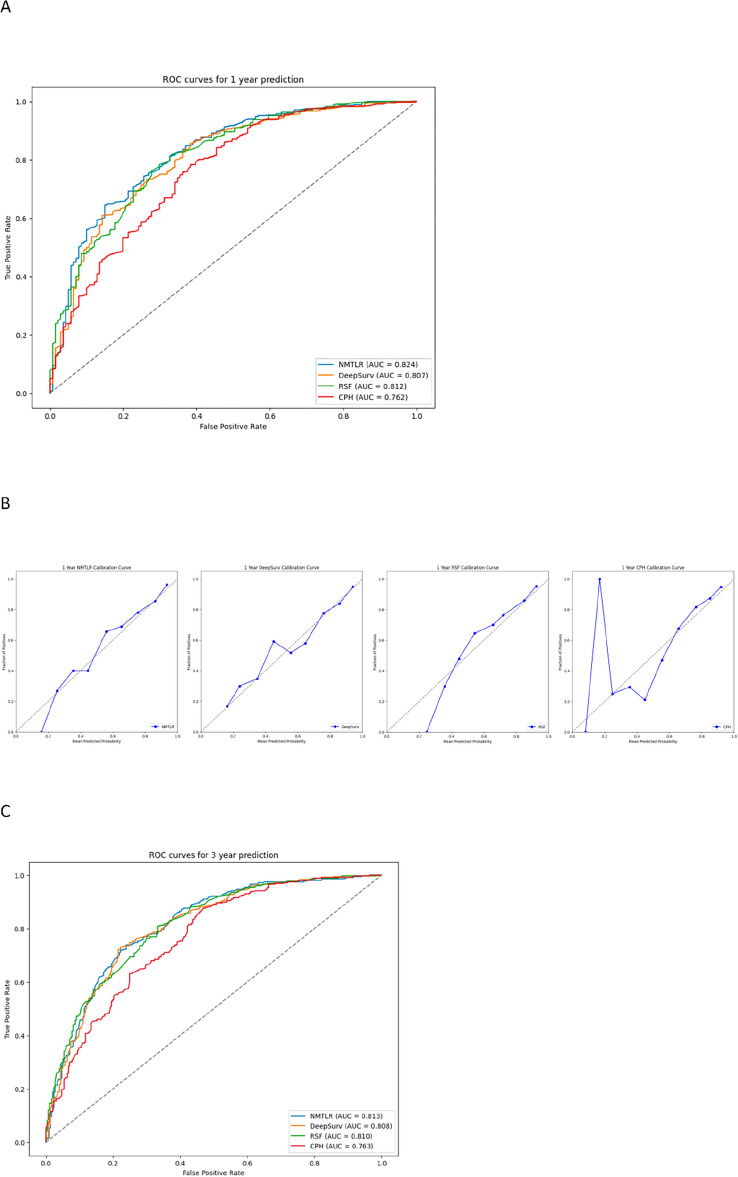

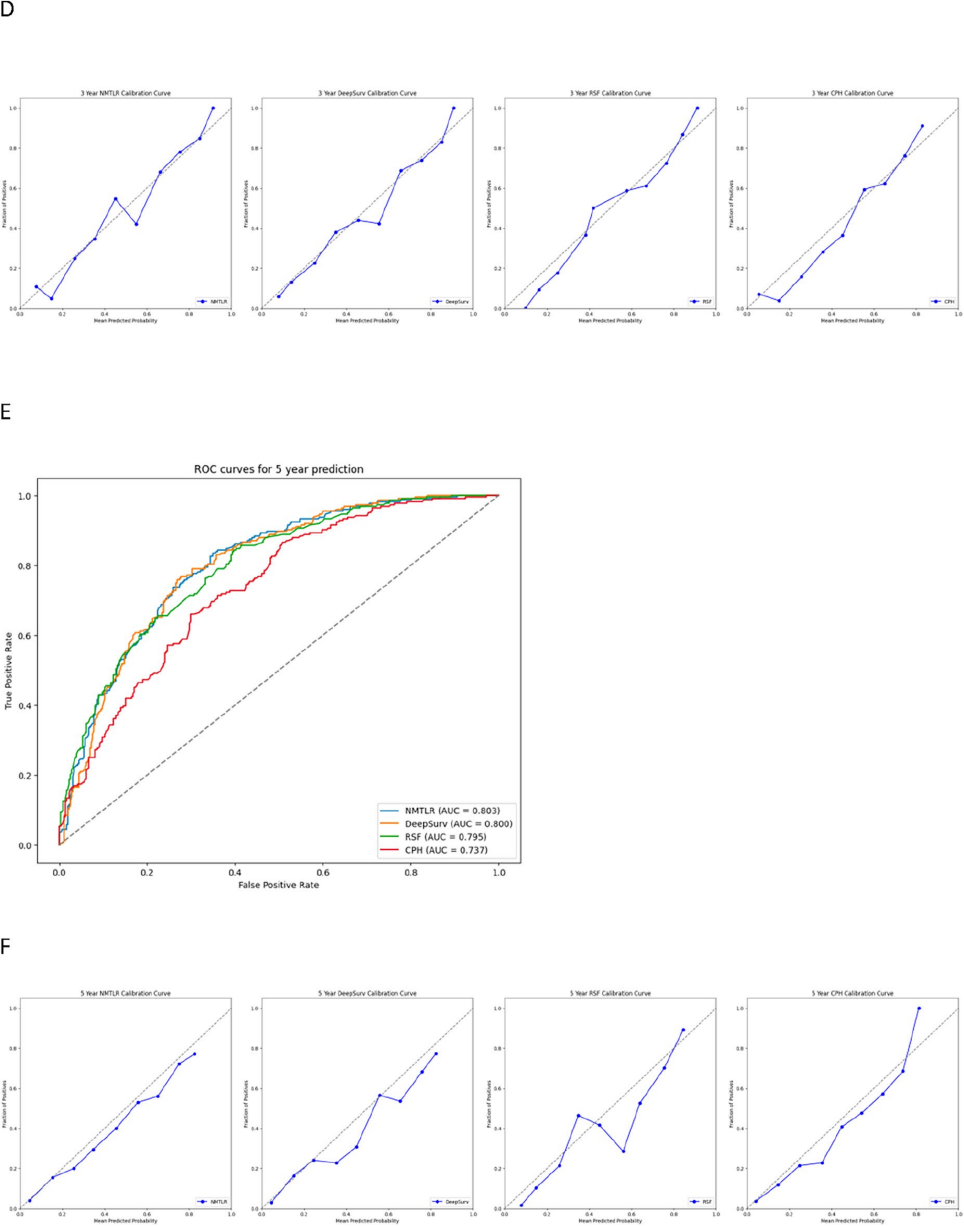
The receiver operating curves (ROC) and calibration curves for 1-, 3-, 5-year survival predictions. ROC curves for (**A**) 1-, (**C**) 3-, (**E**) 5-year survival predictions. Calibration curves for (**B**) 1-, (**D**) 3-, (**F**) 5-year survival predictions.

Through calibration plots (Fig. 4), we observed that the NMTLR model demonstrated the best consistency between model predictions and actual observations in terms of 1-year, 3-year, and 5-year overall survival rates, followed by the DeepSurv model, RSF model, and CoxPH model. This consistency was also reflected in the AUC values: for the prediction of 1-year, 3-year, and 5-year survival rates, the NMTLR and DeepSurv models had higher AUC values than the RSF and CoxPH models. Specifically, the 1-year AUC values were 0.803 for NMTLR and 0.794 for DeepSurv, compared to 0.786 for RSF and 0.766 for CoxPH; the 3-year AUC values were 0.808 for NMTLR and 0.809 for DeepSurv, compared to 0.797 for RSF and 0.772 for CoxPH; the 5-year AUC values were 0.819 for both DeepSurv and NMTLR, compared to 0.812 for RSF and 0.772 for CoxPH. The results indicate that, in predicting the survival prognosis of patients with hepatocellular carcinoma, the deep learning models—DeepSurv and NMTLR—demonstrate higher accuracy than the RSF and the classical CoxPH models. The NMTLR model significantly exhibited the best performance in multiple evaluation metrics.

### Model feature importance

**Figure 5:**
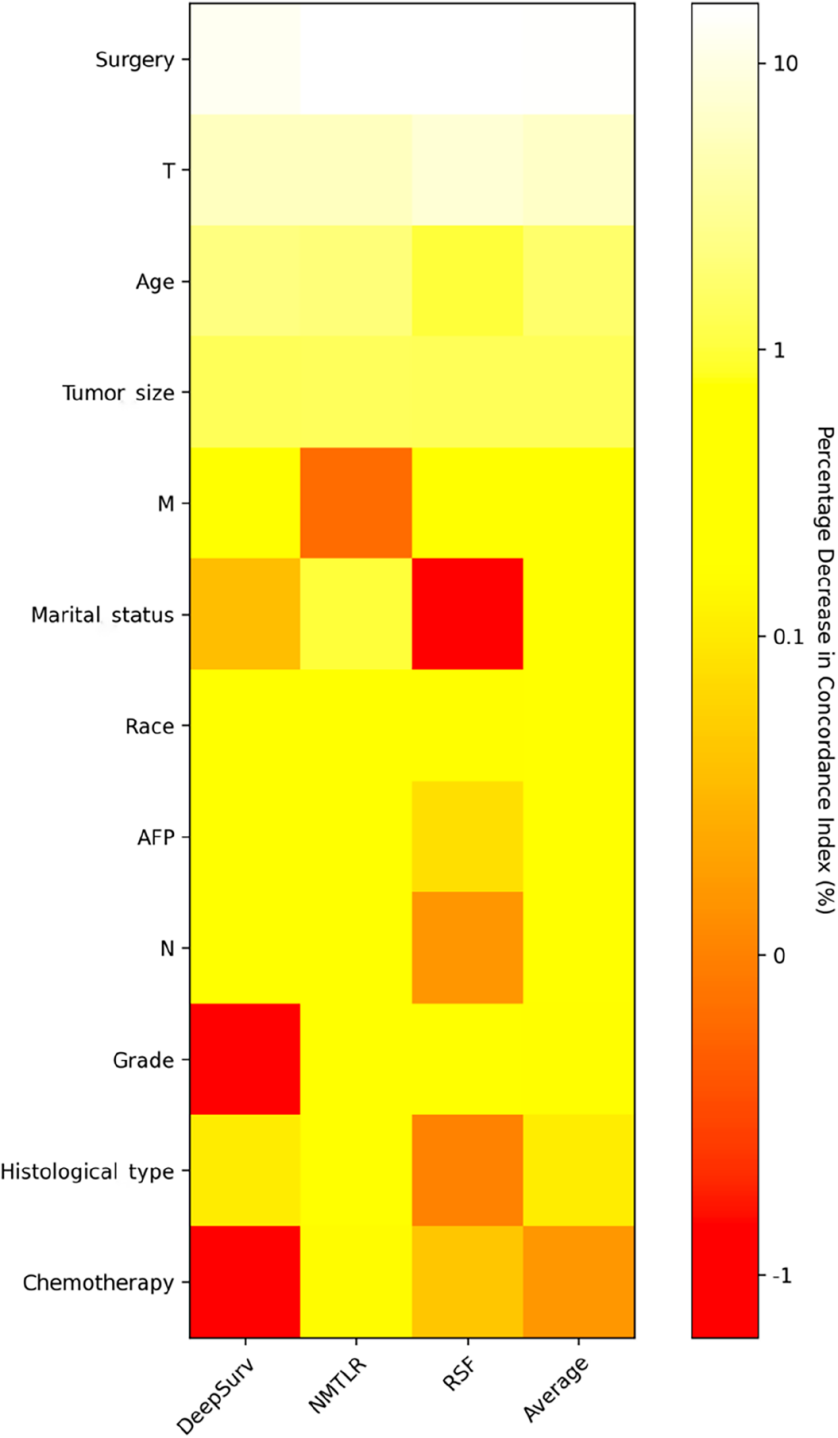
Heatmap of feature importance for DeepSurv, neural network multitask logistic regression (NMTLR) and random survival forest (RSF) models.

In the feature analysis of deep learning models, the impact of a feature on model accuracy when its values are replaced with random data can be measured by the percentage decrease in the concordance index (C-index). A higher decrease percentage indicates the feature's significant importance in maintaining the model's predictive accuracy. Figure 5 shows the feature importance heatmaps for the DeepSurv, NMTLR, and RSF models.

In the NMTLR model, the replacement of features such as age, race, marital status, histological type, tumor grade, T stage, N stage, alpha-fetoprotein, tumor size, type of surgery, and chemotherapy led to an average decrease in the concordance index by more than 0.1%. In the DeepSurv model, features like age, race, marital status, histological type, T stage, N stage, alpha-fetoprotein, tumor size, and type of surgery saw a similar average decrease in the concordance index when replaced with random data. In the RSF model, we found that features including age, race, tumor grade, T stage, M stage, tumor size, and type of surgery significantly impacted the model's accuracy, as evidenced by a noticeable decrease in the C-index, averaging a reduction of over 0.1% when replaced with random data.

### Risk stratification capability of the NMTLR model

**Figure 6:**
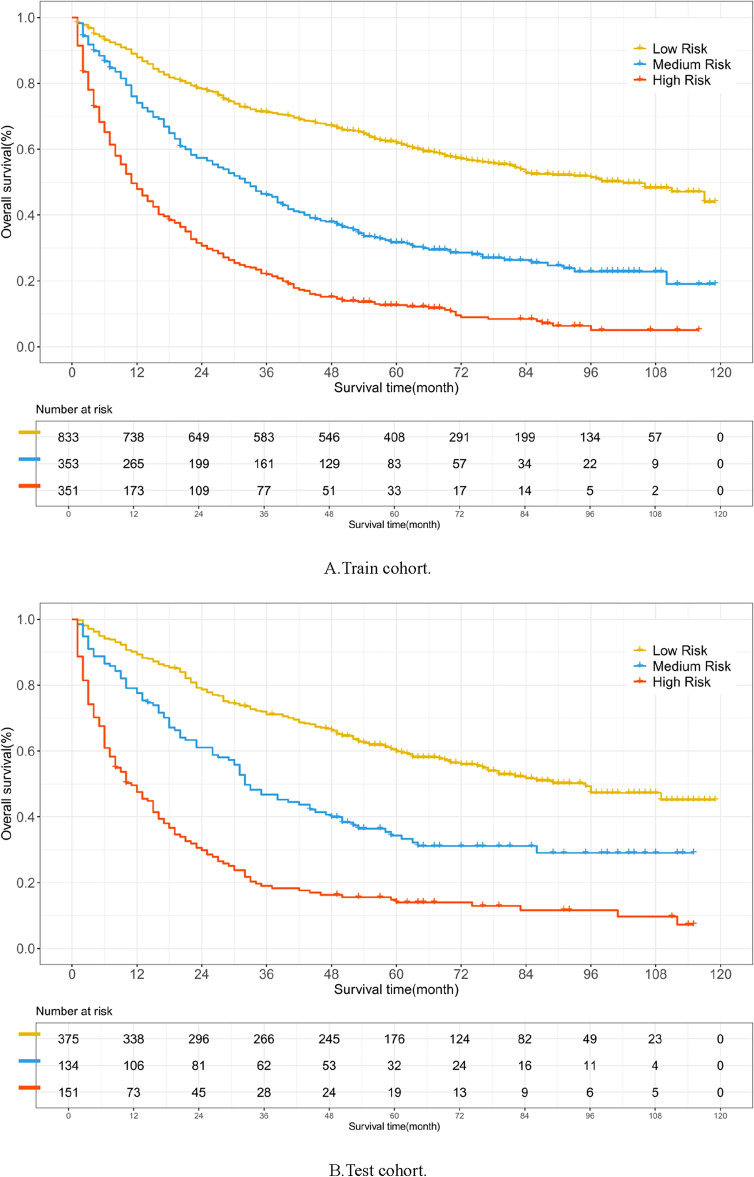
Kaplan–Meier curves evaluated the risk stratification ability of NMTLR model.

In the training cohort, the NMTLR model was employed to predict patient risk probabilities. Optimal threshold values for these probabilities were determined using X-tile software. Patients were stratified into low-risk (< 178.8), medium-risk (178.8–248.4), and high-risk (> 248.4) categories based on these cutoff points. Statistically significant differences were observed in the survival curves among the groups, with a p-value of less than 0.001, as depicted in Fig. 6A. Similar results were replicated in the external validation cohort, as shown in Fig. 6B, underscoring the robust risk stratification capability of the NMTLR model.

### Model deployment

**Figure 7:**
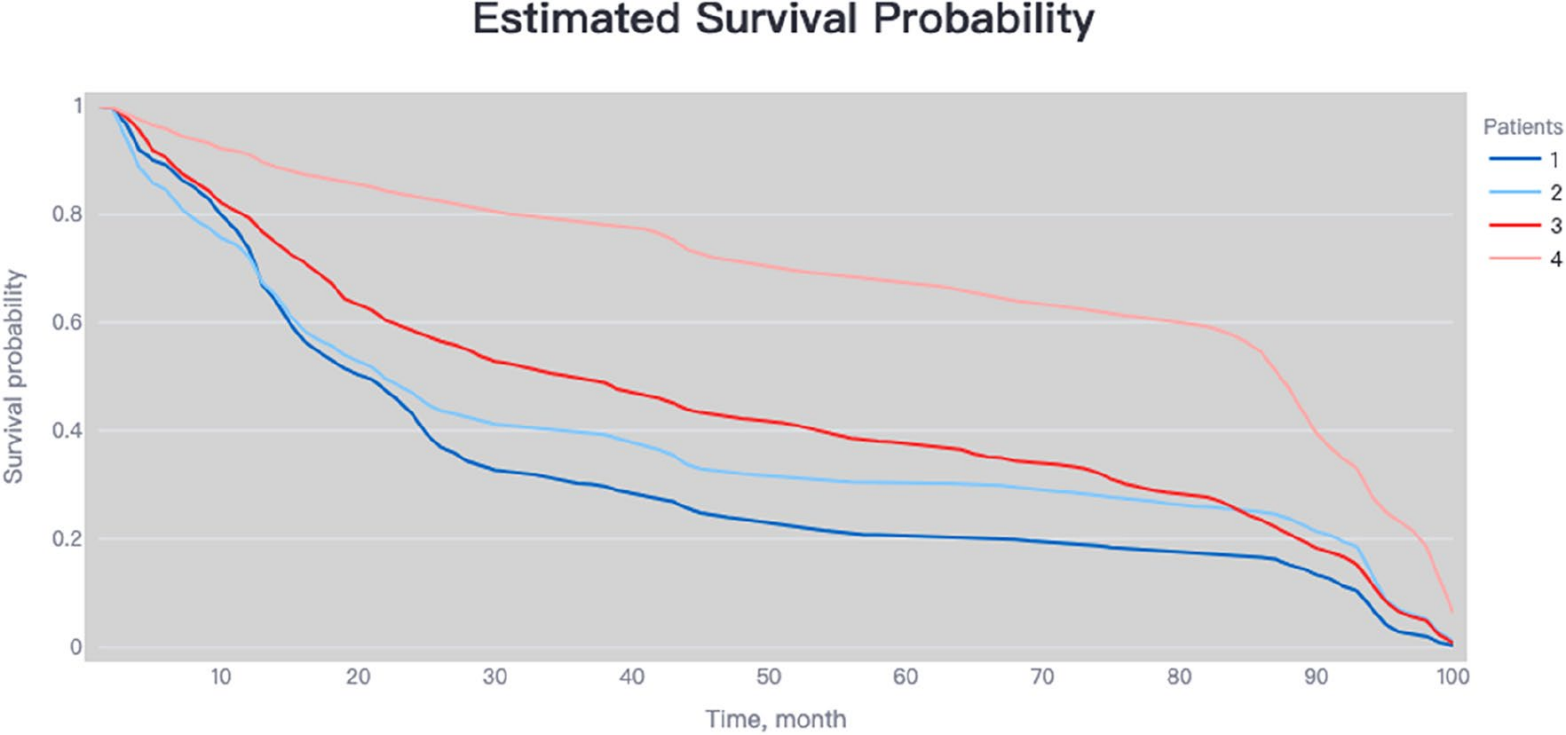
The online web-based application of NMTLR model.

The web application developed in this study, primarily intended for research or informational purposes, is publicly accessible at http://120.55.167.119:8501/. The functionality and output visualization of this application are illustrated in Fig. 7 and eFigure 1 in the Supplement.

## Discussion

Accurately predicting the survival rate of hepatocellular carcinoma (HCC) patients is crucial for their treatment planning and follow-up. Historical studies have revealed various prognostic factors affecting the survival time of HCC patients, including age, tumor size, histological type, tumor grade, metastatic status, and HBV infection^18^. Researchers have developed various models to improve prediction accuracy, such as the BCLC staging, COX proportional hazards model, and RSF^14,19,20^. However, the traditional CoxPH model’s limitations become apparent when dealing with real-world clinical data, particularly in its assumption of a linear relationship between death risk and variables^21^. Thus, deep learning algorithms have started to show their advantages. These algorithms can reveal complex non-linear relationships between factors, hence widely used in survival prediction. This enables a more comprehensive and precise prediction of the survival expectancy of HCC patients. Recent studies have delved into radiomic and genomic data of HCC patients to more accurately identify liver cancer and predict survival rates, achieving significant progress^22–25^. Therefore, we constructed two deep learning models to predict the survival rate of HCC patients and compared their performance with two classic prediction models.

Firstly, we performed statistical descriptions and COX regression analysis on 2197 patients extracted from the SEER database to determine risk factors affecting their prognosis. Eventually, we chose factors such as age, race, marital status, histological type, tumor grade, T stage, N stage, M stage, alpha-fetoprotein, tumor size, type of surgery, and chemotherapy to build the model. In the analysis presented in Table 4, the three machine learning models—DeepSurv, N-MTLR, and RSF—demonstrated significantly higher discriminative abilities compared to the traditional CoxPH model. This result suggests that these machine learning models are potentially more effective in handling complex survival analysis tasks, particularly in scenarios involving high-dimensional data or non-proportional hazards. The superior performance of these models could be attributed to their enhanced ability to capture nonlinear interactions and complex relationships within the data, which are often present in clinical datasets but may not be adequately modeled by traditional methods like CoxPH. Further analysis of the Integrated Brier Score (IBS) reveals that the NMTLR model demonstrates the lowest IBS value (0.1598), outperforming the DeepSurv, RSF, and CoxPH models. A lower IBS value indicates reduced uncertainty in the prediction outcomes, marking an important metric for assessing the quality of model predictions. This underscores the NMTLR model's superior accuracy in forecasting patient survival outcomes. The calibration plots further confirm the superior consistency of the NMTLR model in predicting overall survival rates at 1-year, 3-year, and 5-year intervals compared to observed outcomes. This consistency is also reflected in the Area Under the Curve (AUC) values, an important metric that measures a model’s ability to predict survival at various time points. The NMTLR and DeepSurv models exhibit higher AUC values than both the RSF and CoxPH models at all considered time points. Specifically, the AUC values at 1-year, 3-year, and 5-year intervals are notably superior in the NMTLR and DeepSurv models, highlighting their enhanced performance in predicting the prognosis of hepatocellular carcinoma patients. The NMTLR model performed best in all machine learning models, suggesting its potential application value in clinical practice. These findings provide valuable scientific evidence for further improving the prognosis prediction of HCC patients and advancing precision medicine. By comparing the differences in feature importance among the three models (DeepSurv, NMTLR, RSF), we can see that although each model differs in data processing and prediction methods, certain essential features like age, race, tumor size, T stage, and type of surgery show significant importance in all models. This indicates that regardless of the model used, these features are key factors affecting the accuracy of prognosis prediction in primary liver cancer patients. The NMTLR model predicts patient risk probabilities within the training cohort, effectively stratifying patients into low-risk (< 178.8), medium-risk (178.8–248.4), and high-risk (> 248.4) groups. This stratification not only provides a quantitative estimation of patient risk but also serves as a practical tool to assist in clinical decision-making. Moreover, statistically significant differences in survival curves between any two groups (p < 0.001) are demonstrated in Fig. 6A. This indicates the high efficacy of the NMTLR model in risk stratification, clearly differentiating between patients with varying levels of survival prognosis. Additionally, identical results were obtained in the internal validation cohort, as shown in Fig. 6B, further validating the generalizability and stability of the NMTLR model.DeepSurv and NMTLR models demonstrated superior performance in predicting the survival rate of HCC patients. To apply these models in real-world scenarios, we deployed the two deep learning models into a web-based application, which can be freely accessed via [http://120.55.167.119:8501/]. Through this web application, doctors and medical professionals can conveniently use these deep learning models to make personalized predictions of the survival rate of HCC patients. This will help doctors formulate precise treatment plans and conduct more effective follow-up observations.

Our study still has certain limitations. Some critical information such as chemotherapy type, medication kind, patients' psychological status, religious beliefs, education level, and family cancer history were not fully collected in the SEER database, which might affect the accuracy of predicting the survival rate of HCC patients. Additionally, the data of this study only came from some regions of the United States and did not use external data to validate the prediction models, limiting their universal applicability. Future studies could incorporate data from broader regions and longer-term follow-ups, including patient data from other countries, to further improve the predictive accuracy and relevance of the models. The prognosis of HCC patients is a long-term and complex process, and our study data only covered a period after the patients' diagnosis. Therefore, longer-term follow-up data is crucial for accurately assessing patients' survival rates and prognosis. This will help validate and update the prediction models more comprehensively to reflect patients' actual situation better.

Additionally, although the two deep learning models demonstrated specific predictive capabilities in this study, their black-box nature limits our complete understanding of their computational processes and constraints, posing new challenges for future research. Overall, the outcomes of this study are still subject to factors such as data quality and completeness, and the generalizability of the models needs to be verified on a broader range of datasets. To optimize the performance of the models, future studies might consider incorporating more types of data, such as genomics and proteomics, to enhance prediction accuracy and explore how to integrate predictive models with existing treatment strategies for personalized treatment. This will provide substantial scientific evidence for the predictive assessment and precision medicine of HCC patients.

### Supplementary Information

Supplementary Information 1.

Supplementary Information 2.

## Data Availability

The original contributions presented in the study are included in the article, further inquiries can be download from https://github.com/shouchenghu/HCC.
