# Supplementary material for: Deep learning models for predicting the survival of patients with hepatocellular carcinoma based on a surveillance, epidemiology, and end results (SEER) database analysis
Source: Sci Rep. 2024 Jun 9;14:13232. doi: 10.1038/s41598-024-63531-9 (PMC11163004; doi:10.1038/s41598-024-63531-9)
Supplement: Supplementary file 1 — Supplementary Information 1. [file 41598_2024_63531_MOESM1_ESM.pdf]

eFigure1 The online web-based application of NMTLR model

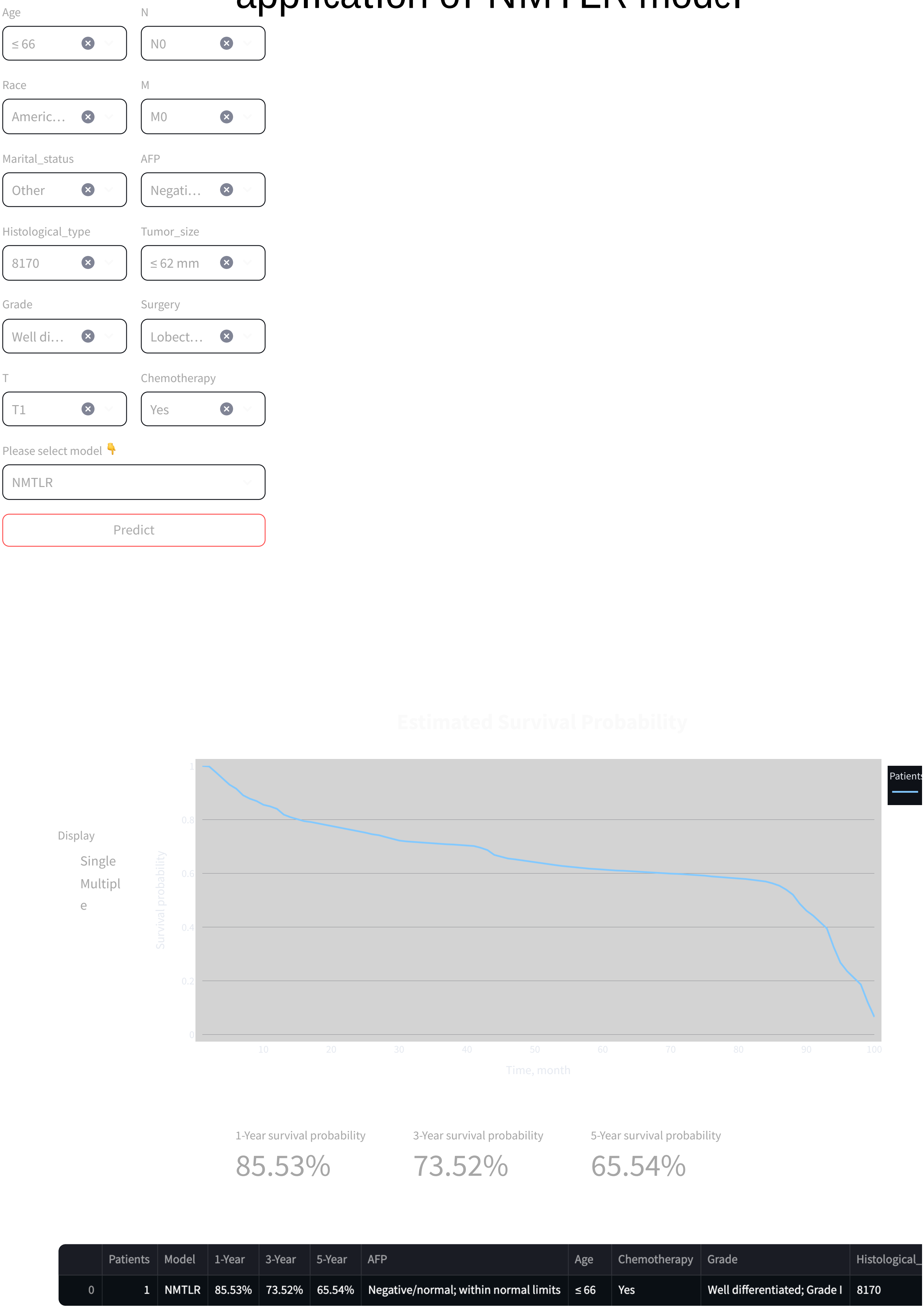

Made with Streamlit
